# 

**Published:** 1885-08

**Authors:** 


PAGE.

ORIGINAL COMMUNICATIONS.

Fœtus Papyraceus. J. H. Etheridge ---- 113
Shock and Nervous Influence in Parturition. H. P. Newman ---- 121
Diet and Management of Artificially-Fed Children. A. H. Foster ---- 131
Syzygium Jambolanum (Jambol) in Diabetes. C. E. Clacius ---- 138
A Case of Leio-Myom of the Vagina and Uterus. H. T. Byford ---- 142
Too-often Feeding of Children. A. E. Hoadley ---- 144
Quadruple Pregnancy. M. Arthur ---- 151

EDITORIAL.

The International Medical Congress of 1887 ---- 153
Ex Pede Herculem ---- 159

DOMESTIC CORRESPONDENCE.

Digitalis ---- 162

SOCIETY REPORTS.

Chicago Gynecological Society—June 19th. Fœtus Papyraceus. J. H. Etheridge ---- 166
A Case of Leio-Myom of the Vagina and Uterus. H. T. Byford ---- 170
Chicago Medical Society, July 20th. Membranous Occlusion of the Posterior Nares. W. E. Casselberry ---- 172
Sanitary Convention at Ypsilanti, Michigan, July 1st, 1885 ---- 176
The Moral Effect of Sanitation. Woodruff ---- 177
Sanitary Needs of School Buildings and Grounds. George ---- 177
Sanitation in Small Cities. Whelan ---- 178
Disposal of Slops and Garbage. Kellogg ---- 179
Limitations and Duties of Local Boards of Health. Whitman ---- 180
Sources of Malaria in Ypsilanti. Kinne ---- 181
Prevention of Communicable Diseases. Wight ---- 181

PAGE

Water Supply. Vaughan ---- 182
The Present Condition of the Water-Supply of Ypsilanti. Shepard ---- 182
The Future Water-Supply of Ypsilanti. Bellows ---- 183
Management of Earth-Closets. French ---- 183
Relations of Sewerage and Water-Supply to the Death-rate in Cities. Smith ---- 183

STATE BOARDS OF HEALTH.

*The Illinois State Board of Health* ---- 185
Licenses Revoked ---- 185
Other Cases Considered, without Final Action by the Board ---- 186
Sanitary Condition of Illinois ---- 186
Sanitary Condition of Chicago ---- 186
Cases of Small-Pox, and of Glanders ---- 186–187
*Michigan State Board of Health* ---- 188
Plans for the Addition to the Reform School, at Lansing ---- 188
Charges by a Legislative Committee, 188
Work of Other State Boards of Health ---- 189
Typhoid Fever ---- 189
Transportation of Infected Articles and Dead Bodies ---- 190
Resume of Work Done by the Board during the Quarter ---- 192

BOOK REVIEWS.

The Curability of Pulmonary Phthisis. Jaccoud ---- 193
A Treatise on Asiatic Cholera. Wendt ---- 195
Elements of Practical Medicine. Carter ---- 196
A Treatise on Practical Chemistry, etc. Clowes ---- 196
The Illinois Anatomical Law ---- 197

BOOKS RECEIVED ---- 200

PAMPHLETS RECEIVED ---- 201

EXTRACTS.

Practical Sanitation ---- 203

